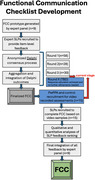# Development of the Functional Communication Checklist for Primary Progressive Aphasia

**DOI:** 10.1002/alz70857_102508

**Published:** 2025-12-25

**Authors:** Jeanne Gallée, Jade Cartwright, Maya L. Henry, Aimee R Mooney, Brielle C Stark, Anna Volkmer, Connie Nakano, Rob J Fredericksen, Kimiko Domoto‐Reilly, Paul K Crane

**Affiliations:** ^1^ University of Washington School of Medicine, Seattle, WA, USA; ^2^ University of Washington Alzheimer's Disease Research Center, Seattle, WA, USA; ^3^ University of Tasmania, Hobart, TAS, Australia; ^4^ University of Texas at Austin, Austin, TX, USA; ^5^ Oregon Health & Science University, Portland, OR, USA; ^6^ Indiana University, Bloomington, IN, USA; ^7^ University College London, London, United Kingdom; ^8^ University of Washington, Seattle, WA, USA

## Abstract

**Background:**

Functional communication (FC) is an indispensable feature of daily functioning and is predictive of patient autonomy and care partner well‐being. Concrete quantitative measures to assess FC in neurodegenerative conditions are lacking. Language‐led conditions such as primary progressive aphasia (PPA) require easily implementable and minimally burdensome FC assessment. This work describes the development of the first instrument to document FC for people living with PPA. The Functional Communication Checklist (FCC) will be developed by incorporating feedback from field experts, partners in research, and item‐level validation.

**Method:**

The FCC is intended to document FC within a setting representative of occupational and community‐based interactions. Initial candidate FCC items were generated by a panel of PPA experts and refined by expert speech‐language pathologists (SLPs) using an electronic Delphi consensus process (see Figure 1). SLP experts were identified and recruited through the International Speech‐Language Therapist/Pathologist PPA Network or via FC publication record. In each Delphi round, participants were asked to rank existing FCC candidate items and provide additional or alternative items. We incorporated feedback from each round to refine the evolving FCC. We provided feedback summaries along with the evolving FCC for each Delphi round. Once no further refinement is suggested, we will evaluate validity and inter‐rater reliability by having 15 additional SLPs use the FCC on PPA assessment videos.

**Results:**

A total of 67 experts have contributed to the FCC development thus far. The current iteration of the FCC evaluates 55 aspects of discourse, social‐pragmatics, language, speech, and cognition. Each domain is evaluated on the spectrum of strength or interference on communication in the context of a short conversational prompt. Background, instructions, conversational examples and item‐level definitions have also been developed through the Delphi process.

**Conclusions:**

The FCC combines clinician observation, patient self‐report, and partner insight to quantify FC and contributing strengths and interferences of speech, language, and nonverbal communication. The quantitative outcomes will facilitate a common taxonomy of FC, enabling interdisciplinary collaboration and consistency across evaluators and sites. The FCC is made possible by partners in research across disciplines and perspectives.